# Fabrication of PLGA nanoparticles with a fluidic nanoprecipitation system

**DOI:** 10.1186/1477-3155-8-18

**Published:** 2010-08-13

**Authors:** Hui Xie, Jeffrey W Smith

**Affiliations:** 1 Sanford-Burnham Medical Research Institute, 10901 North Torrey Pines Road, La Jolla, CA 92037 USA

## Abstract

Particle size is a key feature in determining performance of nanoparticles as drug carriers because it influences circulating half-life, cellular uptake and biodistribution. Because the size of particles has such a major impact on their performance, the uniformity of the particle population is also a significant factor. Particles comprised of the polymer poly(lactic-co-glycolic acid) (PLGA) are widely studied as therapeutic delivery vehicles because they are biodegradable and biocompatible. In fact, microparticles comprised of PLGA are already approved for drug delivery. Unfortunately, PLGA nanoparticles prepared by conventional methods usually lack uniformity. We developed a novel Fluidic NanoPrecipitation System (FNPS) to fabricate highly uniform PLGA particles. Several parameters can be fine-tuned to generate particles of various sizes.

## Background

Particles comprised of the polymer poly(lactic-co-glycolic acid) (PLGA) are widely studied as therapeutic delivery vehicles because they are biodegradable [1] and biocompatible [2-4]. In fact, microparticles comprised of PLGA are already approved for establishing sustained release of leuprolide (Lupron Depot) and triptorelin (Trelstar). Similar PLGA particles also show promise as a delivery vehicle for proteins [5,6], siRNA [7], and for presenting antigens to dendritic cells for vaccination [8-10]. It is also becoming clear that PLGA particles offer considerable flexibility in choosing a route of delivery because they have proven to be effective when injected intramuscularly [11,12], when delivered via inhalation [13-15], and recent results indicate that they also have promise for oral delivery of drugs and antigens [16-19].

Particle size is one of the key features in determining performance because it influences circulating half-life, cellular uptake and biodistribution [20-22]. The kinetic aspects of drug release are also strongly influenced by particle size [23-25]. Early interest in drug-loaded particles centered on their application as vehicles for sustained drug release, but now there is great interest in using similar particles for targeting the delivery of drugs to specific tissues, vascular beds, and cells. For the latter application smaller particles, particularly those in the range of ~100 nm, are likely to be advantageous because they are taken up by cells at rates 15 to 250 fold greater than micron size particles [26]. This difference in the rate of uptake can be the distinction between specific and non-specific uptake. For example, PLGA nanoparticles targeted to dendritic cells with an antibody are taken up specifically, but microparticles targeted with the same antibody are taken up non-specifically [8]. The uniformity of the particle population is also a significant factor in performance. Preparations of particles that are highly uniform will exhibit more consistent biodistribution, cellular uptake, and drug release. Preparations of particles lacking uniformity will exhibit variance in all of these parameters, making it difficult to draw conclusions about which subset of the particle population is responsible for biological effect.

There are many different methods of fabricating solid polymeric particles. Gas flow focusing [27] and electrospray [28,29] can be used to fabricate PLGA microparticles with uniform sizes but these approaches have not been widely used to generate nanoparticles. Several solvent-based methods can be used to make polymeric nanoparticles including interfacial polymerization [30], the evaporation of emulsions [31] and nanoprecipitation [32]. In most cases however, these flow based approaches lack precise control at the macro level, so they yield particles with a broad size distribution. Consequently, extra steps such as filtration or centrifugation are required to isolate the population with the desired size [33]. One solution to this problem is the application of microfluidic platforms, which provide extremely precise control over most aspects of the mixing and precipitation process. For example, Karnik *et al. *developed an elegant microfluidic system that precipitates PLGA nanoparticles by focusing the flow of PLGA in organic solvent by two intersecting streams of aqueous solvent [34]. With this approach highly uniform PLGA particles with diameters of less than 50 nm could be fabricated.

The use of microfluidic devices is not without limitations though. As Quevedo *et al. *pointed out, such devices require specialized fabrication procedures and materials that are not widely available, and they can be easily clogged by particle debris [30]. As an alternative, Quevedo *et al*. proposed a rather simple fluidic system capable of establishing flow conditions suitable for production of monodisperse particles [30]. The utility of the device was demonstrated by using the device to enact interfacial polymerization during flow to produce hollow polyamide shells with diameters ranging from 300-800 μm, depending on polymer concentration and flow rates. Here we show that a similar system, without dramatic reductions in dimension, can be applied to enact an entirely different process, nanoprecipitation.

## Results and Discussion

**Figure 1:**
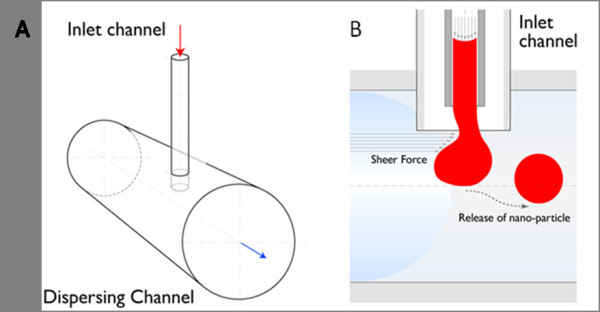
**A schematic of the Fluidic NanoPrecipitation System (FNPS)**. (A) Cartoon of FNPS. Sample inlets are inserted into the dispersing channel. The inlet channel contains PLGA polymer that precipitates upon contact with the surfactant in the dispersing channel, freezing the particles in a spherical morphology. (B) Side view of the channel. PLGA droplets are exposed to the hydrodynamic force of the continuous flow.

Highly uniform PLGA particles with diameters in the range of 140-500 nm, 1000-fold smaller than those generated by Quevedo *et al*., can be generated with the Fluidic Nanoprecipitation System (FNPS). The FNPS can be constructed with general lab equipment and supplies. An inlet channel (26s needle) inserts into the center of a dispersing channel (Tygon tubing with ID 3/32'') (Figure 1). Flow through each channel can be maintained with peristaltic pumps. A major advantage of this flow-based system is that all of the PLGA droplets are created from the end of the inlet channel under precisely the same conditions (e.g. flow rate, injection rate, polymer concentration, *etc*.).

**Figure 2:**
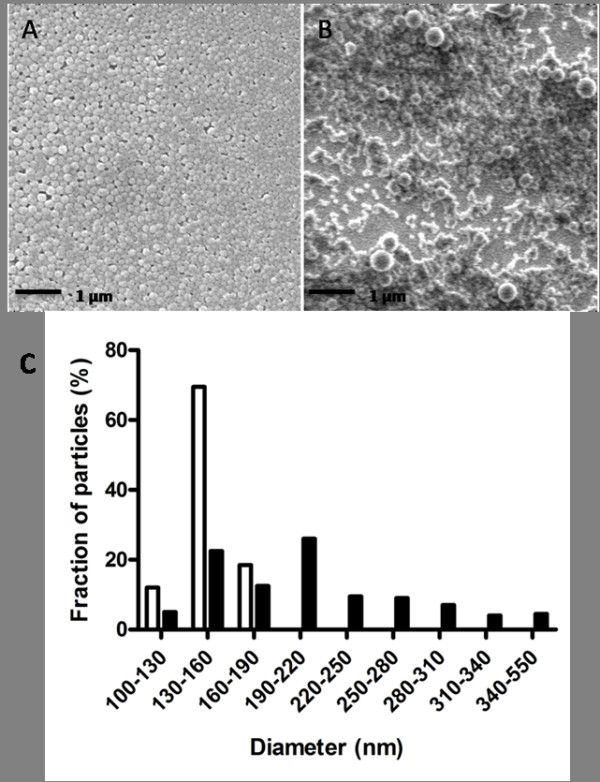
**Highly uniform PLGA nanoparticles are fabricated by the Fluidic NanoPrecipitation System (FNPS)**. Scanning Electron Microscopy (SEM) images of PLGA nanoparticles fabricated by the (A) FNPS, or the (B) conventional nanoprecipitation method. (C) Diameters of the particles were measured by using ImageJ. For each sample, the mean diameter was calculated based on the measurements of 200 randomly chosen particles. White bars indicate the distribution of diameters observed for PLGA nanoparticles fabricated by FNPS (average diameter 148 ± 14 nm). Black bars indicate the distribution of diameters for PLGA nanoparticles fabricated by the traditional nanoprecipitation method (average diameter 211 ± 70 nm). Samples were imaged without prior filtration.

Because the preparation and characterization of well-defined sizes of particles remain a challenge, the performance of this system was gauged by comparing PLGA particles fabricated using the FNPS (Figure 2A) to the conventional nanoprecipitation method (Figure 2B). Particles fabricated by the FNPS have a diameter of 148 ± 14 nm, but particles fabricated by the conventional nanoprecipitation method, using the same solvents and polymer concentrations, are 211 ± 70 nm in diameter. Importantly, the size uniformity of the PLGA particles fabricated using the FNPS is such that all the particles fall within the 100 to 190 nm diameter range, and 70% are between 130 and 160 nm; the particles fabricated using the conventional method have a much broader size distribution, with only 26% having a diameter of 190 to 220 nm (Figure 2C). In order to obtain nanoparticles with small size distribution from conventional nanoprecipitation methods, a filtration step is usually necessary; Gaumet *et al*. reported that as much as 95% of the particles can be lost during filtration [35]. Because of the small size distribution of the nanoparticles generated using FNPS, filtration is not required prior to use.

**Figure 3:**
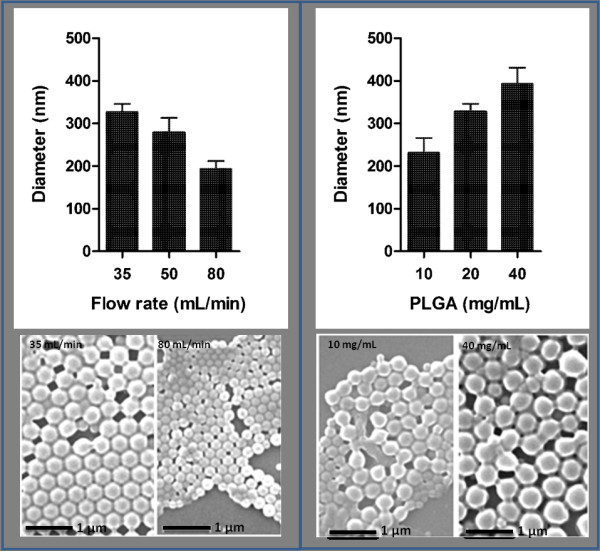
**The diameter of PLGA nanoparticles can be controlled by the flow rates and PLGA concentrations**. (A) SEM images and diameters of PLGA nanoparticles fabricated at dispersing flow rates of 35 ml/min, 50 ml/min, and 80 ml/min. (B) SEM images and diameters of PLGA fabricated at PLGA concentrations of 10 mg/ml, 20 mg/ml, and 40 mg/ml. Diameters were measured by using ImageJ. For each sample, the mean diameter was calculated based on the measurements of 100 randomly chosen particles. Samples are imaged without filtration.

**Figure 4:**
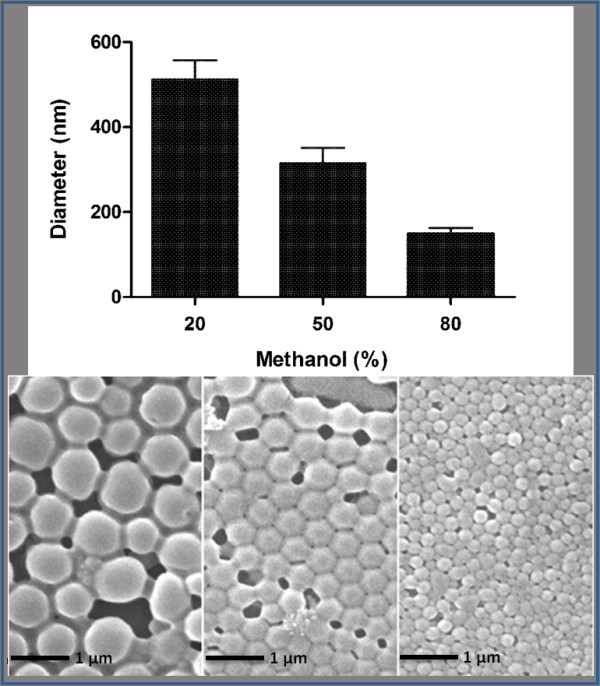
**The diameter of PLGA nanoparticles can be controlled by varying the methanol concentrations (v/v) in the dispersing phase**. Diameter of PLGA nanoparticles fabricated using 20%, 50% or 80% v/v methanol in the dispersing phase of the FNPS. The flow rate of the dispersing channel was maintained at 50 ml/minute. Samples were imaged by SEM without prior filtration. The diameter of the particles was calculated by using ImageJ. For each sample, the mean diameter was calculated based on the measurements of 100 randomly chosen particles.

The size of PLGA particles generated with the FNPS can be changed by adjusting the flow rate of the dispersing phase. For example, a shift from a flow rate of 35 ml/minute to 50 ml/minute and then to 80 ml/minute decreased particle size from 327 ± 19 nm to 278 ± 35 and then to 193 ± 19 nm (Figure 3A). Similarly, a decrease in PLGA concentration from 40 mg/ml to 20 mg/ml and then to 10 mg/ml resulted in a reduction in particle diameter from 393 ± 38 nm to 327 ± 19 nm to 231 ± 35 nm (Figure 3B). Since the FNPS is a water/water miscible solvent system, the composition of the dispersing phase can also be used to control the size of the particles. Increasing the concentration of methanol in the dispersing phase from 20% to 50% and then to 80%, coincided with the reduction in particle size from 512 ± 45 nm to 315 ± 36 nm and then to 148 ± 14 nm (Figure 4). These data suggest that by optimizing all three of these parameters, the FNPS has the flexibility to generate uniform particles across a wide range of sizes from below 100 nm to above 1 μm.

The yield of particles is another important aspect of any fabrication method. We found that the yield of particles from the FNPS is typically 80% of the mass of the PLGA in the inlet solution. Consequently, under the various conditions used for this study, the FNPS generated between two and eight mg of particles/ml/hr. This compares favorably with the yield of three mgs/ml/hr fabricated using similar concentrations of PLGA by the microfluidic system reported by Karnik *et al*. [34]. The FNPS has many advantages including the ability to scale up production by simply increasing the number of inlets entering the dispersing phase. The dispersing stream could also be recirculated to increase the final concentration of particles in the fluid. In addition, because the devise has a low risk of clogging, it can be used continuously.

The mechanism by which the FNPS is able to generate such small and uniform particles is worthy of discussion. One factor that influences the final size of the solidified particles is the size of the monodisperse droplets from which they are precipitated. Quevedo *et al*. [30] demonstrated that the flow in a fluidic system with dimensions similar to that used here is comparable to a traditional microfluidic system. They also found that a higher Reynolds number favors the formation of smaller droplets. So then, parameters like the flow rate in the dispersing channel, and the liquid composition within that channel will impact Reynolds number and can be used to control the size of droplets. These conclusions are entirely consistent with our observation that the flow rate alters the final particle size.

The actual process of nanoprecipitation will also influence particle size. This is how our approach differs from that of Quevedo *et al. *[30]. They used the T-junction system to assist in the precipitation of *emulsions *that were subsequently made solid by *interfacial polymerization *via the action of a cross-linker in the dispersing channel. This process creates "hollow" particles with diameters of several hundred microns. In contrast, we directly precipitated the PLGA polymer by rapid solvent exchange, also called nanoprecipitation [32]. The mechanism of particle formation during nanoprecipitation is not entirely understood, meaning that the precise outcome cannot be predicted. Nevertheless, as has been previously discussed [32], nanoprecipitation appears to be governed by the Marangoni effect, wherein movement in an interface is caused by longitudinal variations of interfacial tension [36]. In such a case, precipitation is driven by i) solute transfer out of the phase of higher viscosity, which is influenced by high concentration gradients at the interface; and ii) by interfacial tension, which, in the case of the FNPS, is determined by turbulence resulting from flow in the dispersing channel. Consequently, the size of the final particle will be influenced not only by features of the dispersing channel related to Reynolds number, but also by factors that influence interfacial tension. These include the polymer concentration, the presence and concentration of surfactant [37], and the nature of any payload that is co-precipitated into the particles [37]. The depth of insertion of the inlet into the dispersing channel might also influence particle size and geometry due to altered turbulence. However, with this prototype FNPS, it was impossible to test this possibility because we could not control the depth of insertion with great precision.

## Conclusions

In summary, the FNPS described here provides an approach to produce very small and highly uniform polymeric particles, in the absence of sophisticated instrumentation or a microfluidic system. The particles are suitable for multiple uses including drug and imaging agent encapsulation.

## Materials and methods

### Materials

PLGA Resomer RG502H was purchased from Boehringer-Ingelheim (Ingelheim, Germany). PLGA sample solutions were prepared by dissolving PLGA in acetonitrile. For example, a 40 mg/ml PLGA solution was prepared by dissolving 40 mg RG502H in 1 ml acetonitrile. Polyvinyl alcohol (PVA, 87%-89% hydrolyzed) was purchased from Sigma-Aldrich. 1% PVA solution was prepared by dissolving 1 g PVA in 100 ml DI water at room temperature and filtered to remove any particulate matter.

### Device fabrication and experimental setup

A Fluidic NanoPrecipitation System (FNPS) was fabricated by inserting a stainless steel needle (Hamilton HA-91039 26s syringe needle) with an inner diameter 0.11 mm, into a Tygon^® ^tubing (ID 3/32', OD 5/32') that was used to pass the dispersing phase. The needle was inserted to the interior at 50% of the tubing diameter.

The PLGA solution fed into the dispersing channel with a 3 ml syringe controlled by a single syringe pump (KDS100, KD Scientific, Massachusetts, USA). A stream of surfactant (1% PVA solution, 20 ml) passing through the dispersing channel (Tygon^® ^tubing with ID 3/32', and OD 5/32') was controlled by a Fisher Scientific Variable-Flow Peristaltic Pump.

Nanoparticles were prepared starting with 10 and 40 mg/ml of PLGA RG502H polymers in acetonitrile. Samples (0.2 ml) were injected at a flow rate of 3.2 μl/min. Nanoparticles were collected into a beaker for analysis. The nanoparticles were washed by centrifuging for 15 minutes using an Eppendorf 5415R at 13200 rpm at room temperature and then removing the supernatant. The nanoparticles were resuspended in DI water by bath sonication (Branson's Model B200). This was repeated three times and the final suspension was sent for analysis.

### Scanning Electron Microscope (SEM)

SEM experiments were conducted by depositing the nanoparticle suspension on freshly cleaved mica and allowing them to dry. A thin film of Au was sputtered onto these mica substrates with sample. Samples were imaged with scanning electron microscopy (SEM; JEOL 5800LV) without filtration or purification. Particle size was measured by using ImageJ. For each sample, the mean diameter was calculated based on the measurements of 100 randomly chosen particles.

## Competing interests

The authors declare that they have no competing interests.

## Authors' contributions

JWS and HX conceived and designed the experimental strategy and interpreted the findings.

HX performed all experiments. All authors read and approved the final manuscript.
